# The adrenergic system in cardiovascular pathophysiology: a translational science point of view

**DOI:** 10.3389/fphys.2014.00356

**Published:** 2014-09-17

**Authors:** Giuseppe Rengo

**Affiliations:** Scientific Institute of Telese Terme, Istituto di Ricovero e Cura a Carattere Scientifico, Salvatore Maugeri FoundationTelese Terme, Italy

**Keywords:** GRK2, heart failure, sympathetic nervous system, beta-blockers, beta-adrenoceptors, functional recovery, exercise training

Heart failure (HF) is one of the leading causes for mortality and morbidity worldwide. Despite advantages in the management and treatment of this syndrome, nowadays, it is estimated that 50% of HF patients die within 5 years from diagnosis (McMurray et al., 2012). Thus, a better understanding of the molecular mechanisms underlying structural, functional, neuro-hormonal, and metabolic alterations of the failing heart is necessary for the identification of new therapeutic targets and strategies. The adrenergic system is crucial for cardiac function, and even more critical in diseased states characterized by elevated sympathetic nervous system (SNS) hyperactivity (Lymperopoulos et al., 2013). SNS hyperactivity is a salient characteristic of chronic HF and causes cardiac up-regulation of G protein-coupled receptor kinase 2 (GRK2), which in turn induces beta-adrenergic receptor dysregulation in the heart (Rengo et al., 2009, 2012).

The present Research Topic aims to present some of the more relevant and recent acquisitions on the molecular abnormalities of the adrenergic system occurring in HF. Dr. Lymperopoulos has reported the molecular mechanisms of regulation of SNS in HF patho-physiology, discussing their therapeutic implications for the failing heart (Lymperopoulos, 2013). The importance of SNS hyperactivity as a main therapeutic target in HF, represents the rationale for the use of beta-blockers, as discussed by Drs. Barrese and Taglialatela. These authors also reported the molecular bases explaining the differences in response to beta-blocker therapy among HF patients (Barrese and Taglialatela, 2013). Dr. Ferrara and collaborators showed the molecular similarities between physiological aging and HF. Both these conditions are characterized by SNS hyperactivity and cardiac beta-adrenergic receptor signaling dysfunction, and this may help to explain why HF is more frequent and its manifestation more severe in the elderly patients (Ferrara et al., 2014). The interconnections between adrenergic system and cardiac metabolism, oxidative stress and nitric oxide signaling have also been discussed in this Research Topic. It is known from several years that the adrenergic system has a profound effect on the regulation of cardiac metabolism. In this regard, Ciccarelli et al. reported the most updated discoveries in the molecular mechanisms involved in the interactions between adrenergic system hyperactivity and metabolic abnormalities, such as insulin resistance and altered glucose metabolism (Ciccarelli et al., 2013). The effects of beta-adrenoceptors on Reactive Oxygen Species generation are described by Corbi et al.; these authors reported also a fascinating hypothesis of the involvement of sirtuins on beta-adrenergic receptors signaling with a potential role in HF pathophysiology (Corbi et al., 2013). Dr. Conti and collaborators reported the mechanisms of the crosstalk between nitric oxide and beta-adrenergic receptor system, in particular in the control of endothelial function and vascular tone (Conti et al., 2013).

Evidences accumulated over the past 20 years support the pathogenic key role of cardiac GRK2 levels/activity in determining HF-related beta-adrenergic receptor dysfunction and cardiac inotropic reserve reduction. All these data indicate GRK2 inhibition, via gene therapy, as a new HF therapeutic approach that has been shown to be compatible and, in some models, also synergistic to beta-blockers. Cannavo et al. provide a contemporary update of this field by describing the therapeutic potentialities of this approach and its beneficial effects not only on beta-adrenergic receptor signaling, but also on cardiac metabolism, apoptosis, and mitochondrial dysfunction (Cannavo et al., 2013). De Lucia and collaborators extended the therapeutic potentialities of GRK2 inhibition to the adrenal glands and to the control of HF-related SNS outflow (de Lucia et al., 2014). Since its first demonstration by Lymperopoulos et al. (2007), GRK2 appeared to be critical in the regulation of adrenal alfa2 adrenergic receptor function, extending also to other organs the therapeutic benefits of GRK2 inhibition in HF. Finally, Dr. Leosco in his review explained the molecular mechanisms involved in the beneficial effects of exercise training in curbing SNS hyperactivity and beta-adrenergic receptor dysfunction observed in HF. A crucial role seems to be played by the ability of physical activity to reduce GRK2 levels both in the heart and in the adrenal medulla, with relevant effects on cardiovascular function (Leosco et al., 2013).

The overall Research Topic indicates that the great advances achieved in the last decades in understanding the molecular alterations involved in the pathophysiology of HF are opening new opportunities for the treatment of this syndrome and, potentially, their future application to the clinical practice might result to further improvements of patient care. Moreover, the interesting new findings, discussed herein, will hopefully stimulate further research on these arguments.

## Conflict of interest statement

The author declares that the research was conducted in the absence of any commercial or financial relationships that could be construed as a potential conflict of interest.
